# Pathological and Epidemiological Assessment of Trematode Burden in Ruminants From Central Ethiopia

**DOI:** 10.1002/vms3.70809

**Published:** 2026-03-27

**Authors:** Adisu Wakuma Boke, Yacob Hailu Tolosa, Abdi Feyisa Fufa, Jirata Shiferaw Abosse

**Affiliations:** ^1^ Department of Clinical Studies College of Veterinary Medicine and Agriculture Bishoftu Ethiopia; ^2^ Department of Microbiology Parasitology and Poultry Health College of Veterinary Medicine and Agriculture Bishoftu Ethiopia

**Keywords:** abattoir, Ethiopia, pathology, ruminant, trematode

## Abstract

**Background:**

Trematode infections in ruminants cause major economic losses through reduced productivity and liver condemnation, with their epidemiology closely linked to snail habitats, rainfall patterns, and poor grazing management. A cross‐sectional study was conducted from November 2022 to March 2023 to characterize pathological changes caused by trematode infections and identify associated risk factors in ruminants slaughtered at three selected abattoirs in central Ethiopia.

**Methods:**

A total of 256 ruminants, 137 bovine, 64 ovine and 55 caprine were included in the study. The sample size was determined based on the number of animals slaughtered per week and the frequency of slaughtering. Haematological analysis, coproscopic examination, and both ante‐mortem and post‐mortem inspections were performed to detect trematode infections.

**Results:**

The overall prevalence trematode was 49.22%, with the prevalence of 32.42% and 16.80% for fasciolosis and paramphistomosis, respectively. There was no statistically significant association between fasciolosis and animal species, but a significant association was found between paramphistomosis and fasciolosis. Fasciolosis prevalence was highest in caprine (41.82%), followed by ovine (31.25%) and bovine (29.50%). For both fasciolosis and paramphistomosis, prevalence was higher in animals with poor body condition (47.62% and 28.57%, respectively), moderate in those with medium body condition (32.81% and 28.97%), and lowest in animals with good body condition (28.97% and 15.89%). Based on geographic origin, the highest prevalence of fasciolosis was recorded in animals from Jimma (52.63%), while paramphistomosis was most common in animals from Dukem (22.50%). Histopathological findings in *Fasciola*‐infected animals revealed haemorrhages, hepatocytic wall dilatation, necrosis, hypertrophy, and portal fibrosis. In *Paramphistomum*‐infected animals, muscular degeneration and loss of villi and microvilli were observed. Haematological analysis showed reduced red and white blood cell counts and elevated liver enzyme levels.

**Conclusion:**

The study revealed a high prevalence of fasciolosis and paramphistomosis in the study areas. Further research is recommended to explore the epidemiology of trematode infections, identify intermediate snail hosts, and develop effective strategic control measures.

## Graphical abstract

• Trematode infections were highly prevalent (49.22%) among ruminants in central Ethiopia, with fasciolosis (32.42%) and paramphistomosis (16.80%) strongly associated with poor body condition and specific geographic origins.

• Histopathological and haematological analyses revealed severe liver and intestinal damage, anaemia, and elevated liver enzymes, highlighting the need for targeted control strategies.

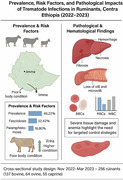

## 1 Introduction

Ethiopia owns the largest livestock population in Africa, comprising an estimated 70 million bovine, 42 million ovine, 52 million caprine, 8 million camels, and 56 million chickens (Central Statistical Agency of Ethiopia 2021). Ruminants are vital to the national economy, contributing around 40% to the agricultural gross domestic product (GDP), 20% to the overall GDP, and another 20% to foreign exchange earnings (World Bank 2017). However, despite this substantial potential, livestock productivity remains low due to multiple challenges. These include parasitic diseases, particularly helminth infections, inadequate nutrition, poor genetic performance, market inefficiencies and weak veterinary and extension services (Benin et al. 2003, Negassa et al. 2011).

Among these constraints, helminth infections, especially those caused by trematodes, pose a major threat to animal health and productivity. Trematodes are internal parasites that infect a broad range of vertebrate hosts, including both animals and humans (Ballweber 2001; Bogitsh et al. 2019; Rubina et al. 2014; Yami and Merkel 2008). They can localize in various organs such as the liver, lungs, intestines and blood vessels, often resulting in significant morbidity and productivity losses (Qian et al. 2016; Walz et al. 2015).

Fasciolosis is among the most widespread trematode infections, affecting bovine, ovine, caprine and occasionally humans. Its transmission depends on the presence of suitable intermediate host snails (usually Lymnaea species) that thrive in wet, marshy or irrigated areas. The disease is endemic in tropical and subtropical regions, particularly where livestock graze near freshwater sources. Infection occurs when animals ingest encysted metacercariae attached to vegetation or in water. Seasonal variations in rainfall and temperature influence snail population dynamics, making fasciolosis more prevalent during and after rainy seasons. It causes significant economic losses due to liver damage, reduced weight gain, poor feed conversion efficiency, infertility, decreased meat and milk quality and even mortality (Yesuf et al. 2020). Young animals are particularly susceptible to adult liver flukes, often experiencing stunted growth and increased vulnerability to secondary infections (Kanyari et al. 2017).

Paramphistomosis, caused by *Paramphistomum* species (rumen flukes), also relies on *aquatic* snail intermediates such as Planorbis or Bulinus species. The life cycle and environmental requirements are similar to those of *Fasciola*, explaining why the two infections often coexist. Immature flukes migrate through the intestinal mucosa before settling in the rumen and reticulum, causing enteritis, diarrhoea, dehydration and reduced feed conversion in heavily infected animals. The disease occurs most commonly in humid and low‐lying regions with abundant snail habitats. This condition is common in tropical and subtropical regions, including Ethiopia (Melaku and Addis 2012), Nigeria (Dube and Aisien 2010) and Thailand (Sripalwit et al. 2007). Although frequently underestimated, paramphistomosis can lead to reduced feed intake and impaired digestion, especially in young or immunocompromised animals.

Dicrocoeliosis, caused by *Dicrocoelium dendriticum* or *Dicrocoelium hospes*, differs from the other two in its transmission ecology. It involves land snails (e.g., *Cionella* spp.) as the first intermediate host and ants (e.g., *Formica* spp.) as the second. Because it does not require aquatic environments, it is common in dry, hilly, or semi‐arid regions where the other flukes are less prevalent. Infection occurs when ruminants accidentally ingest infected ants while grazing (Alian et al. 2021). Adult flukes reside in the bile ducts, leading to chronic cholangitis, fibrosis and liver dysfunction. Both wild and domestic ruminants are susceptible. Economic losses include reduced productivity, organ condemnation, decreased draught power, and in some cases, mortality. Moreover, the zoonotic potential of these parasites poses a public health concern (Hossain et al. 2011; Odigie and Odigie 2013).

Despite the considerable impact of trematode infections on livestock health and production, most abattoir‐based studies in Ethiopia have focused predominantly on fasciolosis. Limited data exist on the prevalence, parasite load, and pathological effects of other trematodes such as *Paramphistomum* and *Dicrocoelium* species. This knowledge gap hampers the development of targeted and effective control strategies, particularly those grounded in comprehensive clinical, pathological and epidemiological evidence.

To help fill this gap, the present study aimed to investigate the clinical, pathological and histopathological effects of trematode infections in ruminants slaughtered at three selected municipal abattoirs in central Ethiopia. Additionally, the study sought to identify key risk factors and examine the relationship between parasite burden and various health indicators.

This study was conducted to investigate the clinical, pathological, and histopathological alterations caused by trematode infections and to identify their associated risk factors in ruminants slaughtered at selected municipal abattoirs in central Ethiopia. Specifically, the study aimed to determine the abattoir‐based prevalence of major trematode infections in slaughtered cattle, sheep and goats, and to identify related risk factors; to characterize the gross and microscopic pathological changes induced by adult trematodes; and to evaluate the relationship between parasite burden and the observed pathological, histopathological, haematological and serum biochemical alterations in infected ruminants.

## 2 Materials and Methods

### 2.1 Study Area

**FIGURE 1:**
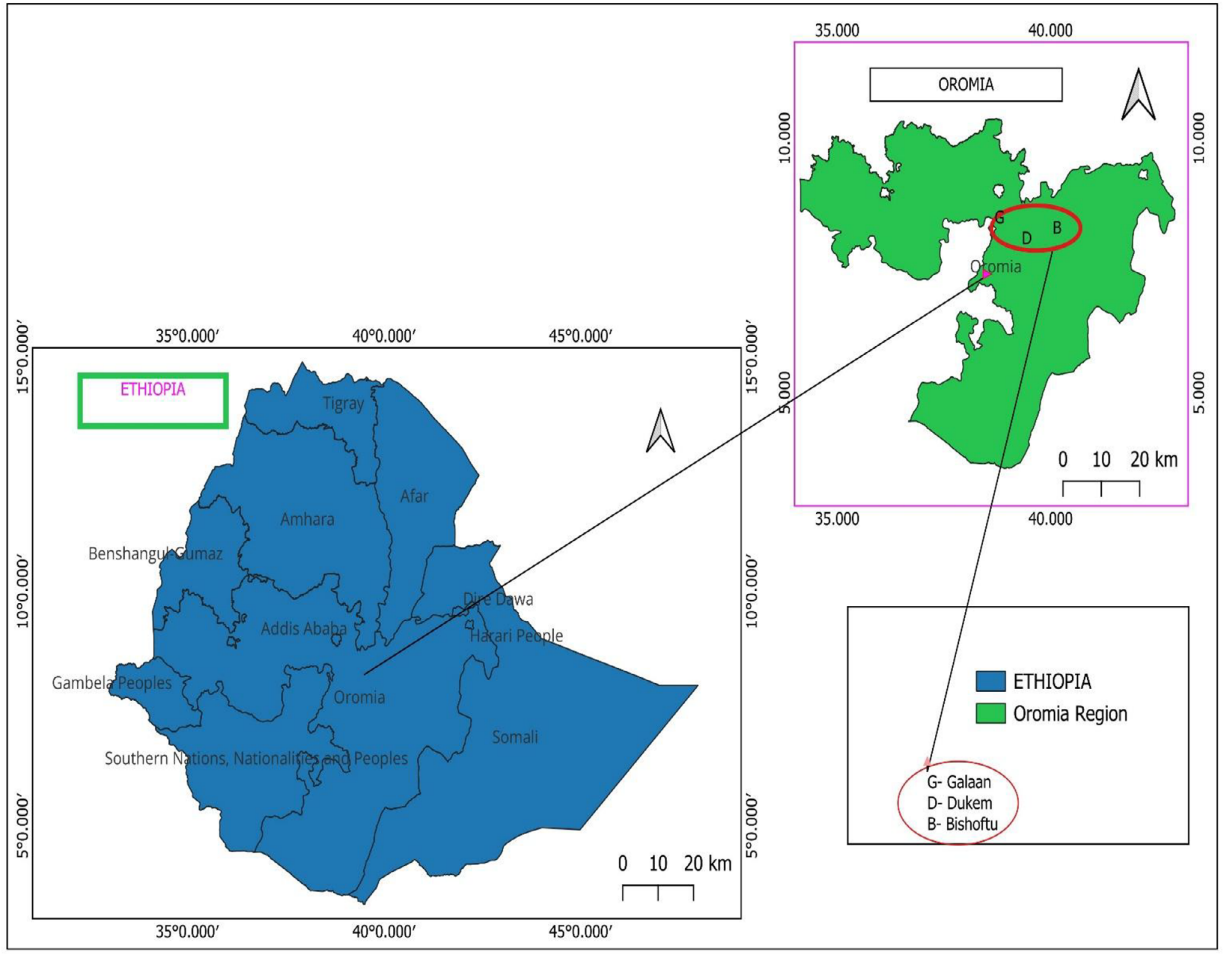
Map of study areas.

This study was carried out at three municipal abattoirs; Bishoftu, Dukem and Galaan, located in the Oromia Region of central Ethiopia, from November 2022 to March 2023. Bishoftu lies at an altitude of approximately 1950 m above sea level and experiences a bimodal rainfall pattern, with a long rainy season from June to October and a shorter one from March to May. The town is situated at 8°45′ N latitude and 38°59’ E longitude, roughly 48 km southeast of Addis Ababa (Central Statistical Agency of Ethiopia 2021). Galaan and Dukem, adjacent towns along the Addis Ababa–Djibouti highway, are located between 1800 and 2300 m above sea level. Geographically, these towns lie between 8°45′ N and 8°53′ N latitude and 38°46′ E to 38°56′ E longitude, about 25 to 37 km south of the capital (Figure 1). The region's average annual temperature is approximately 19°C, with an average rainfall of 861 mm (National Metrological Agency of Ethiopia 2013).

### 2.2 Study Animals

The study involved bovine, ovine and caprine of various age groups and body conditions brought for slaughter to the selected municipal abattoirs. Based on established criteria (Mebrahtu and Beka 2011), the animals were grouped by age: bovines into young (between 2 and 4 years) and adults (> 5 years), and small ruminants (ovine and caprine) into young (< 2 years) and adults (> 2 years). Body condition was also categorized into three groups: poor, medium and good. The characteristics and conditions of the animals were determined during ante‐mortem inspection and based on information from merchants who brought them from different areas. The animals were sourced from various regions, including Gimbichu, Wolaita, Jimma, Akaki, Arsi Negele, Galaan, Hararghe, Adama, Bishoftu, Dukem and Modjo. A total of 256 ruminants were examined: 137 bovine, 64 ovine and 55 caprine, undergoing both ante‐mortem and post‐mortem examinations. The samples were determined based on the animals’ size and frequency of slaughtering per‐week.

### 2.3 Study Design

A cross‐sectional study design was employed from November 2022 to March 2023. Ante‐mortem (blood and faecal samples) and post‐mortem (liver, bile duct, rumen and gall bladder) assessments were performed. A systematic random sampling approach was used to select animals, where the first subject was chosen randomly and every subsequent animal was selected at regular intervals. The prevalence of trematode infection was analysed based on several host‐related risk factors, including species, age, sex, origin and body condition.

### 2.4 Sample Collection and Processing

At ante‐mortem, blood and faecal samples were collected, and during post‐mortem, liver, bile duct, gall bladder and stomach samples were obtained. Post‐mortem examinations involved careful inspection, palpation and incisions of organs to recover adult flukes, following standard meat inspection protocols described by Soulsby (1986). Pathological lesions were characterized, and serum samples were subjected to biochemical analysis. Tissues from affected organs were preserved in 10% neutral buffered formalin for histopathological examination.

#### 2.4.1 Faecal Sample Collection and Examination

Faecal samples were collected directly from the rectum of each selected animal, stored in tightly sealed, labelled sampling bottles and transported to the Parasitology Laboratory at the College of Veterinary Medicine and Agriculture. Samples were examined immediately and refrigerated at 4°C, where analysis was delayed. The sedimentation method described by Antonia et al. (2002) and Bowman (2014) was used to detect *Fasciola* spp. and rumen fluke eggs. *Dicrocoelium* spp. was identified based on the characteristic eggs (Sargison et al. 2012), and egg differentiation between *Paramphistomum* and *Fasciola* spp. followed the procedures by (Urquhart et al. 2003).

#### 2.4.2 Ante‐Mortem and Post‐Mortem Examination

During post‐mortem inspection, organs were labelled based on animal codes. Visual inspection and palpation were followed by systematic incision of the liver, bile ducts and hepatic lymph nodes. For fasciolosis, different lobes of the liver were incised to detect flukes in the parenchyma and bile ducts. Gross lesions and lymph node abnormalities were noted. Organs like the liver, gall bladder, rumen, reticulum and duodenum were thoroughly examined for trematodes, as these are common predilection sites of *Paramphistomum* and *Dicrocoelium* spp. Detected parasites were recorded, and lesions were classified as mature or immature based on their appearance and location (Sohair and Eman 2009).

#### 2.4.3 Tissue Processing and Histopathology

After documenting gross changes, tissue samples from infected animals were fixed in 10% neutral buffered formalin and sent to the Animal Health Institute (AHI) in Sebeta. Samples were processed using an automated tissue processor with increasing alcohol concentrations, cleared with xylene and embedded in paraffin. Sections (5 µm thick) were prepared, dewaxed, rehydrated and stained with hematoxylin and eosin (H&E). The slides were mounted with DPX and examined under a light microscope according to Bancroft and Gamble (2002).

### 2.5 Haematological and Biochemical Analysis

#### 2.5.1 Blood Sample Collection

Blood was drawn from the jugular vein and stored at +4°C in EDTA tubes. All samples were processed within 6 h. Red and white blood cells were manually counted using a haemocytometer. Haemoglobin (Hb) concentration was determined using a haemometer, and haematocrit values were obtained through capillary tube centrifugation (Alexander and Grifiths 1993). Blood smears were stained and examined under oil immersion to determine differential leukocyte counts (Brown 1976).

#### 2.5.2 Haematological Procedures

Haematological analyses were performed following standard procedures. Hb concentration was determined according to the method described by Dein (1984), by comparing the colour of the sample with a standard after the addition of diluted hydrochloric acid. The total erythrocyte count (TEC) was conducted using a 1:200 dilution in Haym's solution, and red blood cells (RBC) were manually counted under a microscope (Philippe 2009). Similarly, the total leukocyte count (TLC) was performed using 0.1 N HCl, with cell counts taken at low magnification (Dein 1984). Packed cell volume (PCV) was measured using microhaematocrit tubes centrifuged at 12,000 rpm for 5 min, and results were compared with standard reference values (Ibrahim and Zerihun 2012). Differential leukocyte count (DLC) was carried out by fixing air‐dried blood smears in methanol, staining them with Giemsa, and classifying 100 white blood cells to determine the percentage of each cell type.

#### 2.5.3 Biochemical Analysis

Blood collected in plain vacutainers was allowed to clot at room temperature before refrigeration. After complete clot retraction, samples were centrifuged at 3000 rpm for 10 min to obtain serum, which was stored at −20°C for analysis (Nasreldin and Zaki 2020). Giemsa staining of blood smears was also performed as per Hodzic et al. (2013).

Serum biochemical tests were used to detect potential organ damage or dysfunction. These included measurements of alanine transaminase (ALT) and aspartate transaminase (AST) to assess hepatocellular injury, as well as alkaline phosphatase (ALP), gamma‐glutamyl transferase (GGT), serum proteins, and bilirubin to evaluate cholestasis and liver function (Boone et al. 2005).

### 2.6 Data Management and Statistical Analysis

Data were entered into Microsoft Excel 2016, coded appropriately, and analysed using STATA version 17. The relationship between trematode infection and risk factors such as animal origin, age, sex and body condition was assessed using chi‐square (χ^2^) tests. Descriptive statistics (frequencies and percentages) were used to summarize pathological and microscopic findings. Haematological values were analysed for mean, range and standard deviation. Logistic regression was applied to evaluate the association between risk factors and abattoir‐level trematode prevalence. Differences between infected and non‐infected animals were compared using a t‐test, with statistical significance set at *p* < 0.05 (SAS 2000).

## 3 Results

### 3.1 Overall Prevalence of Fasciolosis and Paramphistomosis

Among the 256 ruminants (ovine, goat and bovine) at Bishoftu, Dukem, and Galaan municipal abattoirs, 83 (32.42%) and 43 (16.80%) animals were positive for fasciolosis and paramphistomosis, respectively, with a total prevalence of 126 (49.22%) trematodes. The highest prevalence was for caprine 23 (41.82%), ovine 20 (31.25%) and followed by bovine 40 (29.20%). Statistically analysis showed that there was no significant difference among species (*p* > 0.05) (Table 1).

**TABLE 1:** The overall prevalence of fasciolosis and paramphystomosis based variables.

Risk factors	Categories	No. of examined	Fasciollosis (Positive/%)	Paramphystomosis (Positive/%)	_X_^2^	*p* value
Species	Bovine	137	40 (29.20)	10 (7.30)	Fashiollosis (2.91)	0.23
	Ovine	64	20 (31.25)	22 (34.40)	Paramphystomosis (23.40)	0.00
	Caprine	55	23 (41.82)	11 (20.0)		
Origin	Adama	17	2 (11.76)	3 (17.7)	Fashiollosis (10.53) Paramphystomosis (7.84)	0.40 0.64
	Modjo	34	11(32.35)	7 (20.6)		
	Bishofu	49	16 (32.650)	11 (22.5)		
	Dukem	40	13 (32.50)	9 (22.5)		
	Galaan	17	5 (29.41)	3 (17.7)		
	Wolayita	12	5 (41.67)	0 (0.00)		
	Hararghe	5	2 (40.00)	1 (20.0)		
	Arsi Negele	8	1 (12.50	0 (0.00)		
	Gimbichu	27	11 (40.74)	4 (14.8)		
	Jima	19	10 (52.63)	2 (10.5)		
	Akaki	28	7 (28.00)	3 (10.7)		
Age	Young	111	45 (40.54)	17 (15.3)	Fashiollosis (5.90) Paramphystomum (0.31)	0.01 0.58
	Adult	145	38 (26.21)	26 (17.9)		
Sex	Male	214	67 (31.31)	33 (15.4)	Fashiollosis (0.74) Paramphystomum (15.42)	0.39 0.18
	Female	42	16 (38.10)	10 (23.8)		
BCS	Poor	21	10 (47.62)	6 (28.6)	Fashiollosis (2.80) Paramphystomum (2.27)	0.25 0.32
	Medium	128	42 (32.81)	20 (15.6)		
	Good	107	31 (29.97)	17 (15.9)		
	Total	256	83 (32.42)	43 (16.8)		

Abbreviations: *_X_* ^2^, chi square; BCS, body condition.

Among the risk factors considered, the prevalence was highest for animals in young age groups 45 (40.52%), followed by adults 38 (26.21%). Accordingly, fasciolosis prevalence was highest in animals from Jima (52.63%), while the lowest in animals from Adama (11.76%). There was no significant difference in the prevalence of fasciolosis among ruminants based on origin (Table 1). Also, a high prevalence of fasciolosis infections was found in poor body condition animals (47.62%), followed by medium 32.81% and good 28.97% body condition animals. Analysis of the frequency of fasciolosis by species in ruminants was considered in the current study and no statistically significant differences (*p* > 0.05; Table 1).

Paramphistomosis was more common in ovine species (34.38%) than in bovine (7.30%), with a significant correlation between animal species but no significant variation based on origin, sex, age or body condition (Table 1).

### 3.2 Abattoir Survey

A total of 256 ruminants were examined, comprising 137 cattle, 64 sheep and 55 goats slaughtered at the Bishoftu, Galaan and Dukem municipal abattoirs. Among these, 83 animals (32.82%) were found positive for *Fasciola* spp., and 43 (16.80%) for *Paramphistomum* spp. (Table 1). The prevalence in goats (23/55; 41.82%) was significantly higher (*p* > 0.05) than in sheep (20/64; 31.25%) and cattle (40/137; 29.20%).

In terms of species composition, *Fasciola hepatica* was the most prevalent, accounting for 38 cases (45.78%), while *Fasciola gigantica* was the least prevalent, with 27 cases (32.53%). Mixed infections involving both species were detected in 18 animals (21.68%). The overall prevalence estimated in this study is presented in Table 2. The prevalence of *Fasciola* and *Paramphistomum* species was 32.82% and 16.80%, respectively.

**TABLE 2:** Abattoir Percentage of *Fasciola* species in ruminants in selected abattoirs.

*Fasciola* species	No. of infected liver	Percentage (%)
*F. hepatica*	38	45.78
*F. gigantica*	27	32.53
Mixed	18	21.68

### 3.3 Haematological Profile

Haematological analysis showed lower haematocrit, Hb, RBC and WBC results in both fasciolosis and paramphistomosis. A significant association was observed between haematocrit and Hb in ruminants. Differential leucocyte count (eosinophils were higher; neutrophils, monocytes and lymphocytes were lower). A statistically significant association (*p* < 0.05) was observed between erythrocytes in fasciolosis and paramphistomosis (Table 3), there were significant differences between leucocytes in fasciolosis and paramphistomosis (Table 4).

**TABLE 3:** Overall haematological result of mean comparison and trematode infection.

	Infected	Uninfected	
Parameters	Mean ± Std. dev.	Mean ± Std. dev	*p* value
Haematocrit (%)	30.55 ± 8.09	38.5 ± 6.3	0.00
Hb (g/dL)	10.3 ± 3.3	12.7 ± 2.4	0.00
TEC × 10^6^/µL	8.79 ± 40.02	11.7 ± 62.3	0.33
TLE×10^3^/µL	13.3 ± 3.66	70.95 ± 7.23	0.19
MCV (g/dL)	60.69 ± 16.3	60.11 ± 9.74	0.63
MCH (g/dL)	3.31 ± 0.34	3.4 ± 0.65	0.77
MCHC (g/dL)	33.55 ± 6.55	33.08 ± 3.4	0.77

Abbreviations: Hb, haemoglobin; MCH, mean corpuscular haemoglobin; MCHC, mean corpuscular haemoglobin concentration; MCV, mean corpuscular volume; TEC, total erythrocyte count; TLC, total leucocyte count.

**TABLE 4:** Comparative analysis of mean differential leukocyte counts during trematode infection.

	Infected	Uninfected	
Parameters	Mean ± Std. dev.	Mean ± Std. dev	*p* value
Neutrophils	34.09 ± 10.5	37.34 ± 10.2	0.00
Lymphocytes	48.50 ± 9.70	52.30 ± 9.3	0.00
Monocytes	4.44 ± 2.1	5.26 ± 2.93	0.00
Eosinophils	8.47 ± 6.8	7.14 ± 3.0	0.00
Basophils	0.12 ± 0.33	0.3 ± 4.3	0.00

### 3.4 Biochemical Analysis Result

The mean values with the serum biochemical parameters, including ALP, aspartate aminotransferase (AST), alanine aminotransferase (ALT), total protein, and albumin in affected ruminants are presented in Table 5. From the result, the *Fasciola*‐infected ruminants had reduced mean of total protein (4.30 ± 0.49) and albumin (3.97 ± 0.65); however, AST, ALT, and ALP (88.90 ± 5.40, 62.95 ± 11.30, 127.2 ± 12.8) were statistically significant, respectively.

**TABLE 5:** Comparison of serum biochemical analysis in ruminant fasciolosis‐infected and uninfected liver.

		Mean ± SD				
No	Liver condition	AST (μ/L)	ALT (μ/L)	ALP (μ/L)	Total protein (g/dL)	Albumin (g/dL)
1	Uninfected	19.53 ± 3.96	22.33 ± 4.10	75 ± 5.21	7.39 ± 0.92	6.46 ± 0.31
2	Infected	88.90 ± 5.40	62.95 ± 11.30	127.2 ± 12.8	4.30 ± 0.49	3.97 ± 0.65
	*p* value	0.0000	0.0000	0.0000	0.0000	0.0000

### 3.5 Gross Pathological Lesions

**FIGURE 2:**
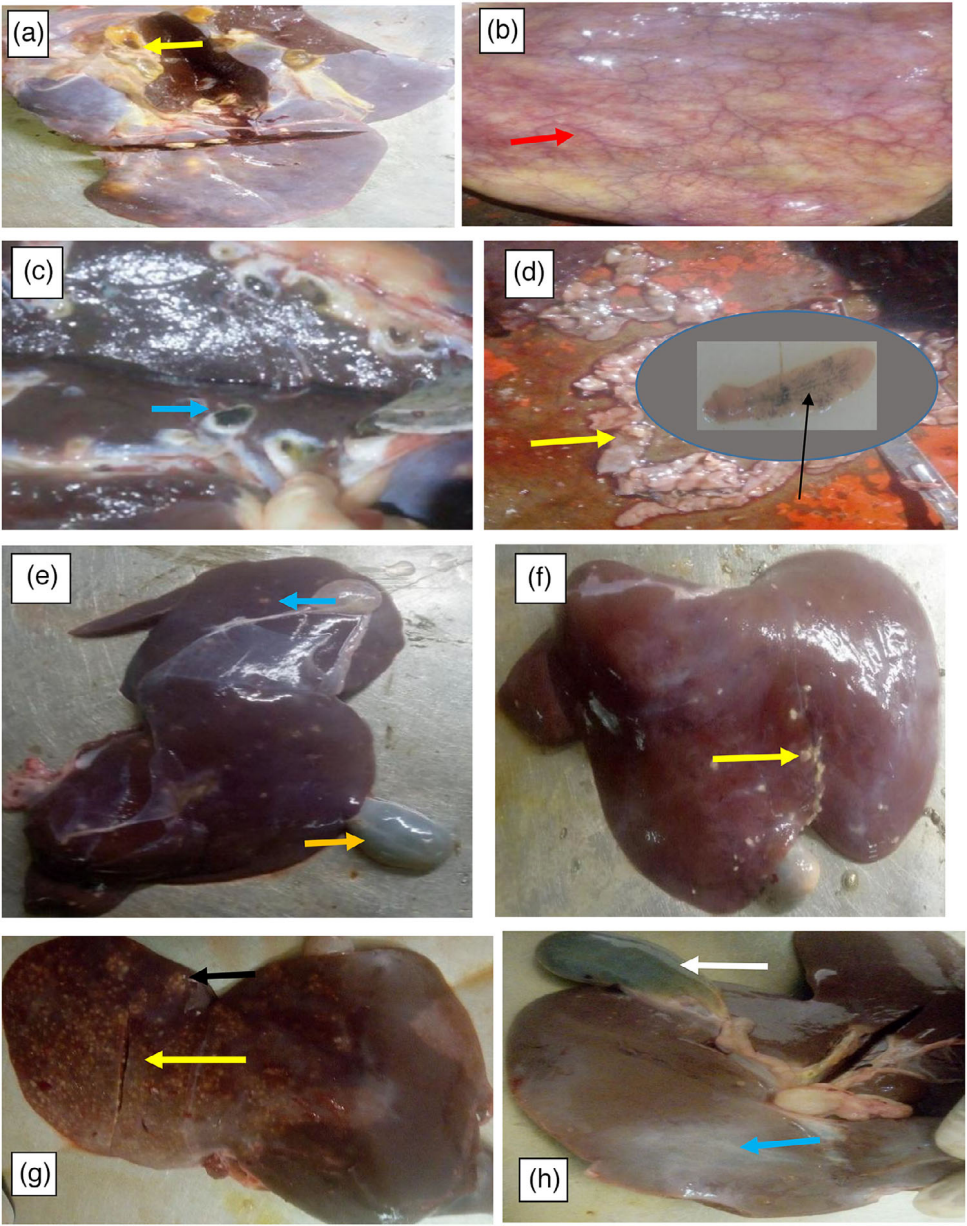
Gross pathology of liver and gall bladder in bovine. (a) Engorgement, hyperplasia of the bile duct and paleness in some areas, which was due to the necrosis (yellow arrow). (b) Cholecystitis (red arrow), (c) heavily infected liver (pipe stem appearance) and bile duct of bovine filled with blackish brown exudates infected with *F. gigantica*. (d) Adult (black arrow) and young (yellow arrow) liver fluke. (e) Fibrosis of the liver (blue arrow) and distended of the gall bladder (yellow arrow) and (f) calcification of liver in caprine. (g) Second stage of cirrhosis and (h) fibrosis (blue arrow), distended gall bladder in ovine (white arrow).

In this study, the most frequently observed gross pathological lesions during post‐mortem examination included firm, pale, swollen livers with irregular outlines and tough texture (Figures 2). Adult liver flukes and calcifications were commonly found within the bile ducts and liver parenchyma. In chronic cases, affected livers appeared smaller in size and exhibited focal to multifocal nodules along with small haemorrhages on the parietal surface. Upon longitudinal sectioning of the bile ducts, abnormal fluke migration paths and evidence of calcification were observed. The bile ducts were often thickened and swollen, containing adult flukes, necrotic debris and signs of cholangitis (Figure 2).

In cases of acute fascioliasis, the liver was markedly enlarged with rounded edges, pale colouration and numerous small to large haemorrhagic spots scattered across the parietal surface. Migratory tracts containing flukes were visible within the parenchyma. Additionally, the hepatic lymph nodes were enlarged, and a thick, cloudy fluid exuded upon sectioning. In some bovine livers, a viscous yellow substance oozed from the cut surface of firm, dark‐brown livers containing multiple soft abscesses surrounded by hyperaemic zones (Figure 2). The gall bladder was often distended due to back pressure from the nodular lesions (Figure 2).

**FIGURE 3:**
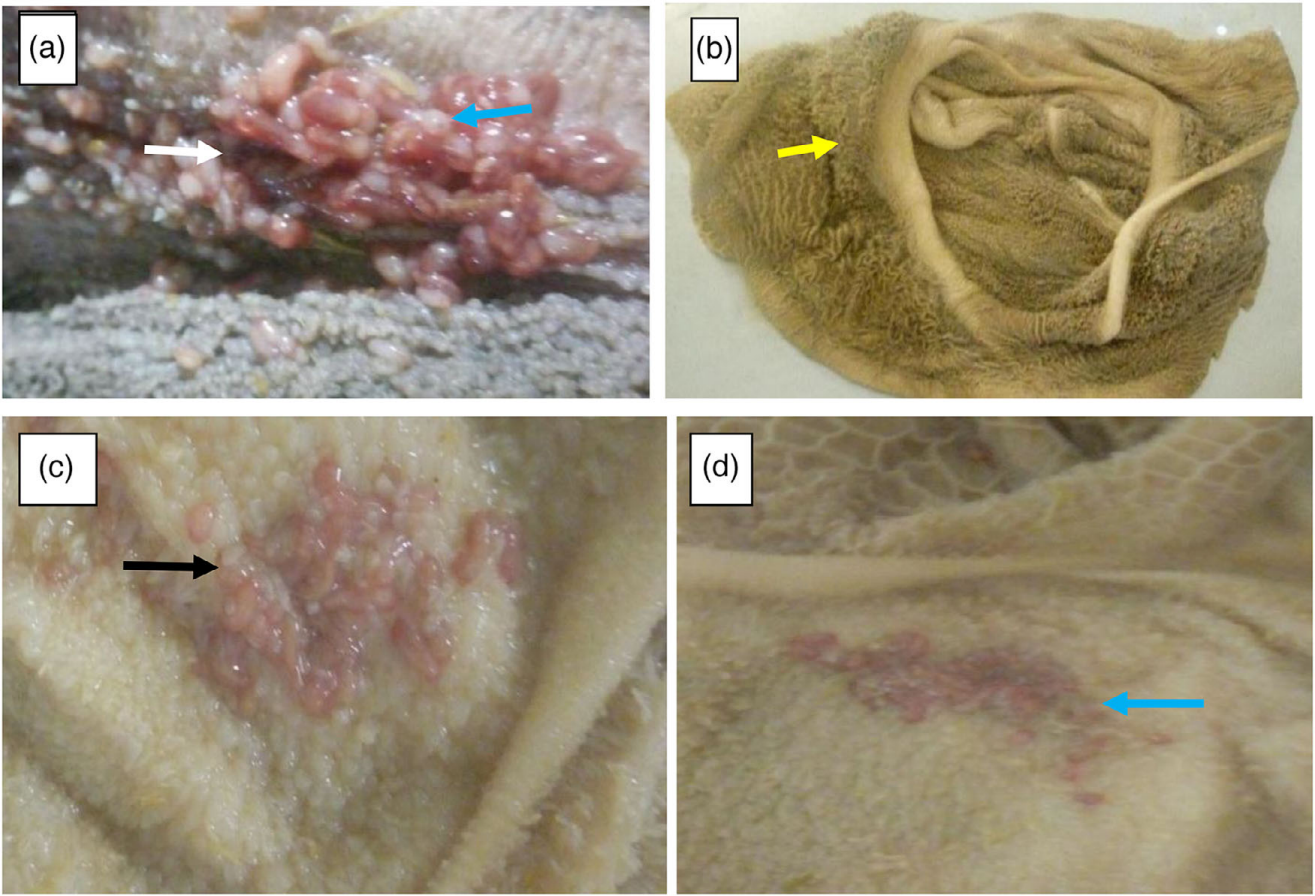
Growth pathology of bovine rumen. (a) Gross pathology of adult rumen fluke attached to rumen papillae in bovine liver. (b) Normal bovine rumen. (c, d) Adult rumen fluke attached to rumen papillae in small ruminants.

Gross pathological changes associated with chronic fascioliasis were characterized by hepatomegaly resulting from parenchymal inflammation and bile duct fibrosis, often with embedded adult flukes. In acute infections, the liver was slightly, too moderately enlarged with rounded edges and paler colouration, and showed extensive haemorrhagic patches over the parietal surfaces of the left, right and caudate lobes (Figure 2). Gross examination of the rumen revealed atrophied and ulcerated ruminal papillae at sites of fluke attachment (Figure 3). The rumen and reticulum were thoroughly washed to facilitate the detection of adult rumen flukes (Figure 3).

### 3.6 Histopathological Results

**FIGURE 4:**
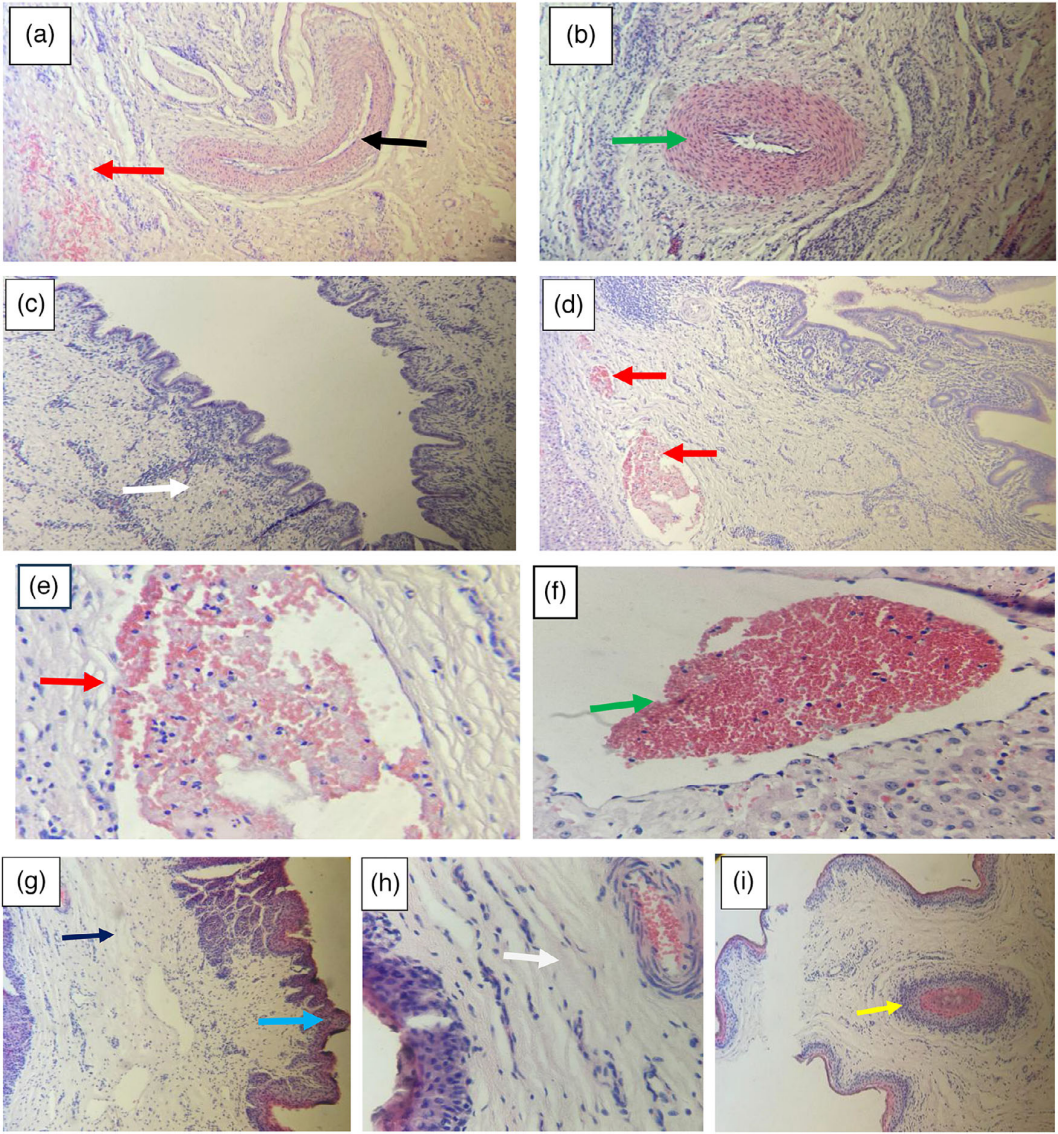
Histopathology of bovine liver with different lesions in bovine. (a) Hypertrophy of central vein with their wall (black arrow), haemorrhages in interstitial spaces and (b) infiltration of eosinophilic cells with thickening of hepatic artery, lymphocytes and few plasmas cells (c) extra hepatic biliary duct hyperplasia, hepatocytes necrotized, infiltration of inflammatory cells (d) haemorrhage, congestion in central vein, infiltration of inflammatory cells, connective tissue multiplication (fibrosis). (e) The RBCs are inside the central vein (f) with congestion and infiltration of inflammatory cells in the central vein. (g) Degeneration of the muscular layer (black arrow), hypertrophy of papillae (blue arrow) of rumen (h) devoid of villi and oedematous region in the glands, (i) granulated regions encapsulated with eosinophils infiltration and externally surrounded by leucocytes (yellow arrow).

Histopathological examination of livers and bile ducts from *Fasciola*‐infected ruminants revealed significant tissue alterations. Common hepatic lesions included haemorrhages within the portal veins, accumulation of purulent exudates, dilation of hepatic sinusoids, and areas of calcification. Hepatocellular necrosis was prominent, with some sections showing hepatocyte hypertrophy, infiltration by eosinophils, and tissue thickening. Portal fibrosis was characterized by fibrous tissue proliferation, infiltration of inflammatory cells, and compressive atrophy of surrounding liver tissue. Bile duct sections exhibited extensive cellular infiltration, fluid‐filled vacuolated areas, epithelial degeneration and wall disintegration (Figure 4).

Similarly, intestinal tissues affected by paramphistomosis displayed marked histopathological lesions. These included haemorrhages in the muscular layers of the rumen, glandular degeneration, loss of villi and microvilli, and disorganization of the muscularis and submucosal layers. Infiltration and degeneration of muscle fibres and submucosa were also observed. Additionally, hypertrophied ruminal papillae were noted, often devoid of villi and associated with oedematous glandular regions. Granulomatous regions encapsulated by eosinophilic infiltration and surrounded externally by leukocytes were also evident (Figures 4).

## 4 Discussion

Fasciolosis is a zoonotic, food‐borne helminth infection caused by flatworms of the Fasciolidae family, predominantly affecting ruminant animals. The chronic form of the disease is the most common and is marked by anaemia, weight loss, liver cirrhosis and impaired hepatic function, often accompanied by atrophy, jaundice and submandibular oedema ‘bottle jaw’ (Ali et al. 2025). This study aimed to identify the three most common trematode parasites affecting domestic ruminants using abattoir‐based meat inspection at three municipal abattoirs in central Ethiopia. A total of 137 bovine, 64 ovine and 23 caprine slaughtered at the Bishoftu, Dukem and Galaan abattoirs were examined. There were no *Dicrocoelium* spp. prevalence reported in this study. The overall prevalence of trematode was 49.62% with fasciolosis across these species was found to be 32.42%. This finding is in line with a study by (Fikirtemariam et al.^2013^), who reported a prevalence of 36.72% in Bahir Dar. However, the current prevalence is lower than that reported by Tolossa and Tigre (2007), who documented a 46.58% prevalence based on post‐mortem liver inspections in Jima and Agaro. Moreover, the observed prevalence of bovine fasciolosis in this study is significantly lower than previous findings by Yilma and Mesfin (2000) (90.65%), Tadele and Worku (2007) (46.58%), and Dejene (2008) (50.98%) at the Gondar, Jima and Arsi abattoirs, respectively.

In the present study, the overall prevalence of paramphistomosis in ruminants was found to be 16.80%. This result aligns with the findings of Buzuwork et al. (2022), who reported a similar prevalence (20.8%) at the Bishoftu municipal abattoir. However, the current prevalence is notably lower than previous reports by Abebe et al. (2011) (57.52% in and around Jima), Melaku and Addis (2012) (40.1% in Bishoftu), and Ayalew et al. (2016) (51.82% at Gondar Elfora Abattoir). These discrepancies in prevalence were due to variations in geographical location, ecological and climatic conditions such as altitude, rainfall and temperature that influence the distribution of intermediate hosts and parasite transmission dynamics. Furthermore, there were lack of routine deworming practices reported for each ruminant species.

This study is also consistent with Keyyu et al. (2006), who found significant differences in parasite prevalence across different age groups. Regarding body condition, the current results indicate no statistically significant association with the occurrence of fasciolosis or paramphistomosis. The prevalence of fasciolosis in animals with poor, medium and good body condition was 47.62%, 32.81% and 28.97%, respectively. For paramphistomosis, the prevalence was 28.57% in poor, 15.63% in medium and 15.84% in good body condition animals. These findings agree with reports by Adi et al. (2023) from the Addis Ababa abattoir and Turuna (2019) at the Nekemte municipal abattoir, who observed higher prevalence in animals with poor body condition. However, this contrasts with the findings of Phiri et al. (2005) and Gojam and Tulu (2018), who reported no significant association between body condition and bovine fasciolosis. Young animals have had fewer exposures and therefore less acquired resistance to *Fasciola*; repeated exposure in adults often produces partial resistance or fibrosis that reduces the establishment of new flukes (Jaja et al. 2017). Sex‐based differences in grazing, watering, housing or anthelmintic treatment schedules (e.g., females left on certain pastures for milk/breeding) can create a higher risk for females in some systems (Jaja et al. 2017).

The study also assessed the origin of the slaughtered animals. Although bovine fasciolosis was more prevalent in animals originating from Jima (52.63%) compared to those from Bishoftu (32.65%), the difference was not statistically significant (*p* > 0.05). The higher prevalence in Jima may be due to favourable environmental conditions that support the survival and proliferation of intermediate snail hosts. No significant association was found between animal origin and the prevalence of paramphistomosis. Variations in sample size, species composition, and management practices may have contributed to these differences. Studies identified that multiple trematodes, including paramphistomes, and identified that some risk factors like breed or body condition are significant, but origin is not always a significant predictor (Aragaw and Tilahun 2018).

Haematological and biochemical analysis revealed reduced levels of PCV, Hb, RBC and white blood cell (WBC) counts, neutrophils, monocytes, lymphocytes, total protein and albumin in infected animals. In contrast, eosinophil counts were elevated. The degenerative and necrotic changes observed in hepatocytes were likely due to elevated plasma concentrations of ALT, a key indicator of hepatocellular damage. This is consistent with findings by Hodzic et al. (2013) and Mbuh and Mbwaye (2005), who reported extensive hepatocyte destruction linked to increased ALT levels in liver fluke‐infected animals. As ALT is primarily localized in the liver, its elevation reflects liver cell death, supporting the conclusions of Kilad et al. (2000), who suggested that raised ALT levels are a consequence of liver damage caused by fluke infection.

Microscopically, infected liver tissues appeared pale and markedly swollen, showing signs of fibrosis. Large fibrotic patches were scattered across the parietal surface, often with a ‘pipe stem’ appearance, indicative of chronic damage. Migration of flukes through liver tissue contributed to severe fibrosis and thickening of the bile ducts (Ali et al. 2024). This pathological form of cirrhosis is the most common in bovine and results from the feeding activity of immature flukes, which inflict direct damage on hepatic cells (Njoku‐Tony and Okoli 2011).

Other consistent histopathological findings included hepatocyte necrosis, haemorrhage, fibrotic changes and increased lobulation of liver tissue. Mononuclear cell infiltration and hemosiderin deposits were observed along fluke migration tracts and portal areas. In ovine naturally infected with *F. gigantica*, granuloma formation around fluke eggs and remnants was also documented. These findings corroborate those of Haroun et al. (2017). (Odigie et al. 2013) also reported enlargement of the liver capsule in diseased animals, which in the current study was associated with the presence of both immature and adult flukes within the bile ducts.

## 5 Conclusion

The study demonstrated the clinical, gross and histopathological changes caused by trematode infections in ruminants, offering important insights into their transmission and pathogenesis. Among the identified parasites, fasciolosis and paramphistomosis were the most prevalent, primarily affecting the liver and rumen, respectively, and leading to significant reductions in body weight. The high infection rates were likely influenced by favourable environmental conditions in the animals’ regions of origin, which promote the survival of intermediate hosts responsible for disease transmission.

Gross and histopathological findings revealed severe parasite burdens, particularly in animals with poor body condition, indicating that infection leads to nutritional deficiencies and blood loss that contribute to weight loss. Although the study intended to examine co‐infections involving *Dicrocoelium* species, none were detected. Overall, fasciolosis and paramphistomosis were found to cause notable economic losses through reduced carcass weight, organ condemnation and tissue damage. The study emphasizes the need for regular deworming, improved pasture management and better housing and feeding practices to control infection, enhance animal health and boost productivity.

## Author Contributions

**Adisu Wakuma Boke**: writing – original draft, methodology, writing – review and editing. **Yacob Hailu Tolosa**: investigation, funding acquisition, validation, project administration. **Abdi Feyisa Fufa**: conceptualization, visualization, software, formal analysis, data curation, writing – review and editing. **Jirata Shiferaw Abosse**: conceptualization, methodology, visualization, supervision, resources.

## Funding

The authors have nothing to report.

## Ethics Statement

Ethical clearance for this research was obtained from the Animal Research Ethics Review Committee of the College of Veterinary Medicine and Agriculture, Addis Ababa University. All animal sampling procedures were conducted in accordance with ethical guidelines under Reference No: VM/ERC/18/03/15/2023.

## Conflicts of Interest

The authors declare no conflicts of interest.

## Data Availability

The authors have nothing to report.
